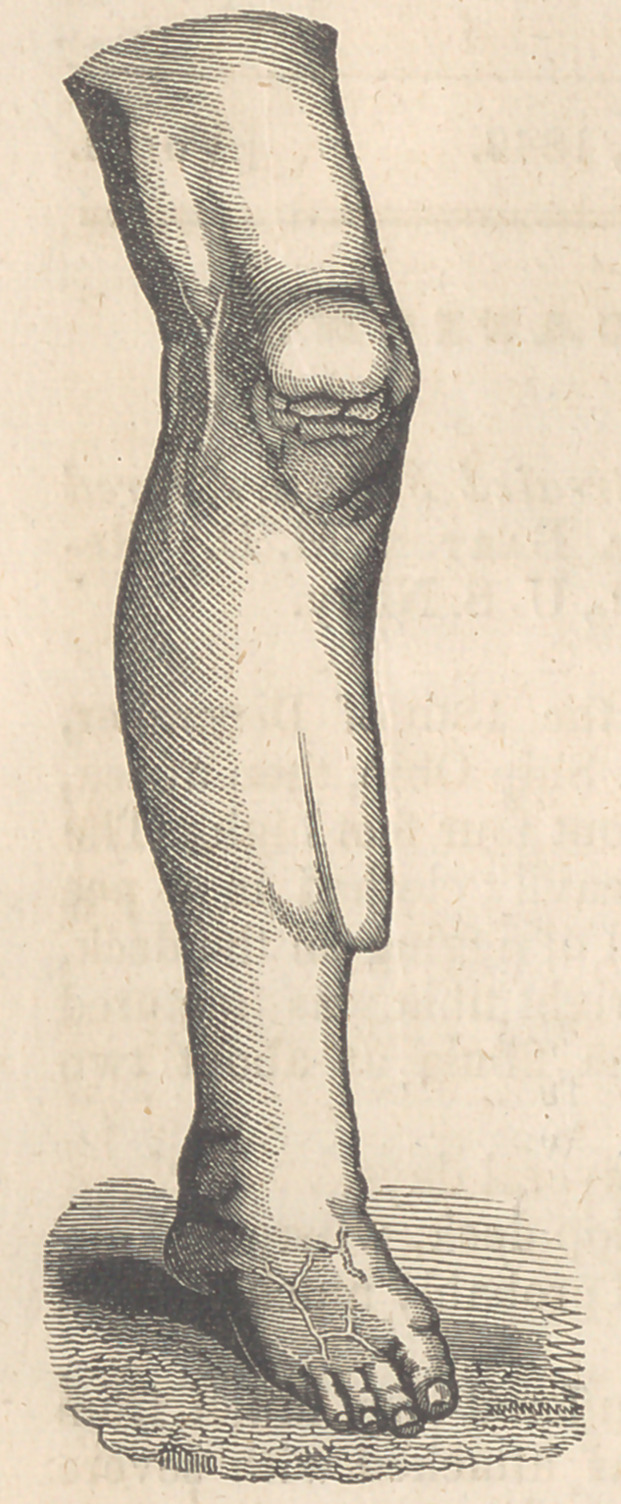# Case of Deformed Leg, from Unsuccessfully Treated Fracture, Cured by an Operation Performed by John Rhea Barton, M. D.

**Published:** 1842-01-08

**Authors:** W. S. W. Ruschenberger

**Affiliations:** U. S. Navy


### MEDICAL EXAMINER.

NEW SERIES.

No. 2.] PHILADELPHIA, JAN. 8, 1842. [Vol. I.

### ORIGINAL COMMUNICATIONS.

*Case of Deformed Leg, from unsuccessfully treated Fracture, cured by an operation performed by* John Rhea Barton, M. D. Reported by W. S. W. Ruschenberger, M. D., U. S. Navy.

About half past seven o'clock, P. M., on the 18th of December, 1838, while in charge of the deck of the U. S. Ship Ohio, then at sea, Lieutenant —— fell from the horse block, about four feet high. The weather being cold and boisterous, he was heavily clothed in a pee jacket, &c. His foot became engaged in a coil of rigging on the deck, while the body was carried forward, and the right tibia was fractured transversely at about its lower third, and the fibula at about two and a half inches above the ankle.

A gale of wind, then commencing, lasted several days.

He was placed in his apartment on the orlop deck, where the circulation of air was very much interrupted, and probably much vitiated by the number of persons breathing it.

The limb was set by the surgeon. He suffered very much from the motion of the ship, and in the night was attacked with severe spasms in the limb; and he distinctly felt the fragments slip upon each other.

On the 5th of January, 1839, the ship arrived at Mahon, and on the following day, the nineteenth after the accident, the patient was moved on shore. During the transportation from the ship, he suffered great pain from the moving of the ends of the broken bones on each other.

He remained in bed eight weeks, and when he got up, the limb was still flexible at the point of fracture. Getting out of bed was at first very painful, and usually occupied fifteen minutes.

When he got up he was urged to exercise the limb, and three or four weeks afterwards, to bear his weight, in a degree, upon it.

In consequence of the accident, and its unsuccessful treatment, the upper fragment of the tibia rides the lower one, overlapping it about half an inch, forming an obtuse angle which presents inwards. The limb is shortened a half inch; there is a concavity inwards, on the outside of the leg, as might be the case if the fibula were pressed inwards against the tibia at its lower third; the flexor muscles are thrown out of their normal line of action, and the external condyle of the femur seems to be, in a measure, alone in the essential constitution of the knee joint, the internal ligaments being elongated, and the knee thrown inwards. *(See the cut.)*

The patient suffers no pain; and the only inconvenience complained of is, that his footing is not certain when at sea, and that he suffers under pain and weakness in the knee on taking unusual exercise on shore.

For the purpose of removing this inconvenience, and correcting the deformity, the patient came to Philadelphia; and after having been carefully examined at different times, and at considerable intervals, by Drs. Thomas Harris, W. E. Horner, J. Randolph, W. S. W. Ruschenberger, J. Rhea Barton, and Paul B. Goddard, anxiously submitted to an operation.

Lieutenant —— is a native of South Carolina; he is thirty-four years of age, about five feet eight inches high, of nervous sanguine temperament, light eyes, ruddy complexion, and, with the exception of an attack of fever on the coast of Africa, in 1824, has enjoyed uninterrupted health. He does not use tobacco in any form.

For a month he has regulated his diet with a view to the operation, eating moderately of meat once a-day.

Having procured an airy, comfortable apartment, and made the necessary preparations, he submitted to the following operation, performed by Dr. J. Rhea Barton, assisted by Drs. Norris, E. Peace, Paul B. Goddard, W. P. C. Barton, and Ruschenberger.

*Oct.* 18th, 1841. Weather clear and cool. Ten minutes before commencing the operation the patient swallowed thirty-five drops of laudanum. He was placed upon the table at twelve o'clock.

Two incisions, three inches in length, were made over, and parallel with the internal and external margins of the tibia, three inches apart at their upper extremities, and two and a half at their termination. These two incisions were connected by a transverse cut, made a little below the nearly square projecting end of the upper fragment of the tibia; the three incisions describing the letter H. The flaps thus formed, consisting of the skin and subjacent cellular tissue only, were raised up, exposing the fragments of the tibia at their point of union. The adjacent muscles were separated from the bone by the handle of the scalpel; the periosteum, very near the lower termination of the upper fragment, was divided by the scalpel: a small saw, somewhat in the form of a carving knife, about ten inches long in the blade, suddenly tapered into a point of two and a half inches long, and rounded at the extremity, was next employed, and a slice of bone, less than a line in thickness, removed from the extremity of the upper fragment. The saw was carefully worked in the same line of direction, and in the same plane, frequently removing it to clear its teeth by a sponge, until the lower fragment was divided nearly through. What remained was forcibly fractured—a short, stout spiculum, adhering to the posterior portion of the lower fragment, and which was afterwards removed.

Upon examination, it was now found, as was anticipated, that transverse bridges of bone connected the tibia and fibula together above and below the seat of fracture,—having been formed there after the injury, for the wise purpose of supporting the weakened limb,—and prevented the upper and projecting portion from being brought in a normal line with the lower fragment. These bony bridges were removed by the aid of a chisel and strong nippers; and, by the same means, the ends of the two fragments of the tibia were adjusted and finally brought into perfect coaptation. The operation occupied nearly an hour. No vessel required ligature; and the loss of blood did not exceed eight ounces.

The edges of the wound were brought together, and retained by adhesive straps. Lint, spread with simple cerate, was placed over them. The limb, from the toes to the knee, was then covered by successive turns of a roller. The patient was now carefully removed to bed. A soft, square pad, two inches thick, was placed over the external maleolus, and a similar one close to the knee joint; upon these was laid a splint two and a half inches wide, to which the two fragments of the tibia were confined by a few turns of a roller, applied at the proper points. A soft pillow, covered with oiled silk, was made to half encircle the limb, by the aid of splints in a splint cloth, and the whole secured by tapes. The limb was protected from the pressure of the bed clothes in the usual way.

Directed to take in the evening a cup of weak black tea, and a piece of dry toast.

9 o'clock, P. M. Patient comparatively comfortable.

19th. 9 o'clock, A. M. Skin natural; very slight suffusion of countenance; bowels not moved, pulse 80, and soft. Had very little sleep since the operation: experienced some few "twinges" in the limb. Became comparatively easy an hour after the operation. Complains of not being able to relax the muscles of the limb. Pain in the back last night and this morning.

A compress placed over the upper fragment, near its inferior extremity, to prevent its rising; it is secured in its place by tying one of the tapes of the splints across it.

Diet.—Barley water, chicken broth.

6 o'clock, P. M. Comfortable. Felt oppressed after taking broth: relieved after passing urine and perspiring freely. Urine brandy coloured.

20th. 9 o'clock, A. M. Pulse 76; skin comfortable; looks cheerful. At 4 o'clock, A. M. had severe continued pain in the abdomen, probably from flatus. Patient applied hot dry cloths with advantage. Tongue slightly white.

Diet.—Small piece of roast beef; barley water.

9 o'clock, P. M. Has not been able to have an evacuation from the bowels from want of convenience, as he cannot get a pan beneath him, and is insurmountably repugnant to the use of a sheet. Complains that he has passed a miserable day: belly very tender and sore, but it is soft and not distended; pulse 76. Moved to a mattrass with a moveable piece on the left side, corresponding to the pelvis. The moving caused great pain and considerable exhaustion.

21st. 9 o'clock, A. M. After taking some warm broth the bowels were moved; and he has had another evacuation this morning. Slept several hours. Soreness and tenderness of the abdomen have disappeared; pulse 76; comfortable; says he is not aware, from any uneasy feeling, that his leg is broken.

External soiled parts of the dressings removed by scissors, and a Scultetus bandage placed over the whole; the oiled silk changed for a clean piece.

Diet continued.

6 o'clock, P. M. Had spasm in the leg in the afternoon.

22d. Half past 9 o'clock, A. M. Pulse 76, and after taking coffee, (which acts kindly on his bowels,) 79. Had very little sleep last night; some spasms; bowels open.

Removed the dressings. Wound healthy.

A number of maggots, nearly a half inch long, about the wound. Washed with Castile soap and warm water. Dressing of lint and simple cerate. Pads at the ankle and knee removed, and the splint, lightly padded, brought into contact with the limb. From a slight thickening of the lower flap, the lower fragment of the tibia *seems* to to be in advance of the upper one.

Continue diet.

6 o'clock, P. M. No sleep; some spasm; great pain in the foot from pressure of the bandages, which was relieved by removing and reapplying them. Wound looks well.

Liquor anodyn Hoffmani. H. S.

23d. Half past 9 o'clock, A. M. Slept about five hours last night; bowels open; pulse 76. Continue treatment.

6 o'clock, P. M. Has had a comfortable day.

24th. Half past 9 o'clock, A. M. Slept four hours; comfortable; pulse 76; bowels open; urine high coloured, but now transparent. Continue treatment.

27th. Nothing remarkable since last report. Slept very little last night. Wash the wound with tinct. myrrh.

Continue diet; half a tumbler of Philadelphia brown stout.

28th. Slept six hours. Bran bags applied. Continue treatment.

*Nov.* 2d. In consequence of the upper fragment having slightly fallen out of its proper line, a straight, narrow splint was applied on the outside of the leg.

5th. Paste board splint applied to the inside of the leg; bone begins to stiffen.

6th. Splint applied on the inside of the leg, which reposes on the pillow without being secured by the splints and splint cloth. Bowels are open every morning; pulse 80; sleeps four or five hours in the twenty-four; is very cheerful.

7th. Wound nearly healed; suppuration has almost ceased.

*Dec.* 31st. At present the patient is walking about his apartment, aided by a cane. He wears the same boots that he wore previous to the operation, and does not think the limb is appreciably shortened. At the end of a fortnight he will be able to travel; but at this time it would be premature to state what the effect of the operation has been, although the limb is straight, and not appreciably shorter than it was prior to the operation.

To the young practitioner of surgery this case is particularly interesting, and should lead all to study carefully the treatment of fractured bones, the result of which, if successful, adds very little to the reputation of the surgeon, but, if otherwise, is calculated to injure his professional character.

The necessary arrangements for variety of matter compel us to defer, until the appearance of our next number, a curious and deeply interesting series of observations on the progress of the operation and the subsequent sensations, made by the patient himself.—*Ed.*

## Figures and Tables

**Figure f1:**